# Application of magnetic fields to the microalgal cultivation of *Nannochloropsis oceanica*

**DOI:** 10.1007/s00449-026-03339-1

**Published:** 2026-04-29

**Authors:** Danielle Barros Urrutia, Bruno Roswag Machado, Pedro Senger de Souza, Carlos Rafael Borges Mendes, Fabio Roselet, Lucielen Oliveira Santos

**Affiliations:** 1 Laboratory of Biotechnology, School of Chemistry and Food, Federal University of Rio Grande, Av. Itália, Km 8, Rio Grande, RS 96203-900 Brazil; 2 MARE – Marine and Environmental Sciences Centre / ARNET – Aquatic Research Network, Faculty of Sciences, University of Lisbon, 1749-016 Lisbon, Portugal; 3 Laboratory of Phytoplankton and Marine Microorganisms, Federal University of Rio Grande, Institute of Oceanography, Av. Itália, Km 8, Rio Grande, RS 96203-900 Brazil; 4 Microbiology Laboratory, Institute of Biology, Health Sciences Center, Federal University of Rio de Janeiro, Rio de Janeiro, RJ Brazil

**Keywords:** Biomass, Microalgae, *Nannochloropsis*, Photoperiod

## Abstract

Microalgae are microorganisms that exhibit variations in their biochemical structures which depend on the species and cultivation conditions. Microalgae produce a variety of biomolecules, including proteins, lipids, carbohydrates, and pigments, the levels of which depend on the species and cultivation conditions. Species that belong to genus *Nannochloropsis,* mainly *Nannochloropsis oceanica,* have been widely used in aquaculture. They are promising sources of biofuels and bioproducts because they produce high contents of proteins and lipids. This study aimed at evaluating the effect of 30 mT magnetic fields (MF) applied to cultivation of *N*. *oceanica* for different exposure periods (1 h d^−1^ and 24 h d^−1^) and photoperiods (12L:12D or 24L). Although no significant differences were observed in the growth rate, the combination of MF applied for 1 h d^−1^ and continuous light (24L) resulted in the highest biomass concentration and lipid accumulation (46.45%). Carbohydrate content ranged from 23.70 to 36.73%, and protein content from 14.80 to 36.43%, indicating that prolonged exposures may reduce proteins. The pigments showed remarkably high levels, especially violaxanthin (0.07 mg g^−1^), vaucheriaxanthin (0.11 mg g^−1^), and chlorophyll a (0.19 mg g^−1^), indicating enhancement of the xanthophyll metabolic pathway and photosynthetic stability. The biomass also exhibited moderate antioxidant activity (16—24%) in the ABTS and DPPH assays. Thus, the study showed that *N*. *oceanica* can grow under different conditions but stands out with 1 h d^−1^ of MF under continuous light as the most promising for enhancing biomass and lipids, with applications in biofuels, food, pharmaceuticals, and nutraceuticals.

## Introduction

Microalgae are unicellular photosynthetic microorganisms which grow under light and CO_2_ and convert them, together with water and nutrients, into biomass that is rich in carbohydrates, proteins, lipids and pigments [1]. They may be grown both outdoors and indoors (controlled growth conditions). Since there is a broad diversity of microalgae, many species have already been registered. The database *AlgaeBase* includes 50,589 species of living algae and 8,081 species of fossil ones, totaling about 61,145 species [2].

Genus *Nannochloropsis* is one that has drawn more interest due to its high production of eicosapentaenoic acid (EPA), which has been used in aquaculture and is relevant to human nutrition [3]. Species *Nannochloropsis oceanica*, which is found in coastal waters and in estuaries, stands out because it can adapt to saline variations, has high productivity and grow in f/2 media [4]. It not only tolerates different osmotic conditions and variations in nutrients and light intensities, fast growth and high lipid content (16–60%) [2–4].

Some studies have shown that photoperiods influence biomass and biomolecule production. Continuous lighting for 24 h can substantially increase biomass productivity, chlorophyll, proteins and carbohydrates, depending on the species and the cultivation system. Combinations of LEDs and thermal stress have already reached 0.75 g L^−1^ biomass and lipid content of 57.6%. A 12L/12D photoperiod favors the synthesis of polyunsaturated fatty acids, such as EPA [8–10].

*N*. *oceanica* has essential fatty acids, vitamins, proteins, carbohydrates and pigments, such as astaxanthin, canthaxanthin, zeaxanthin and chlorophyll [3, 5–11]. To increase their production, magnetic field (MF) application has become a promising technique. Exposure to MF can interact with biological systems, although the effects vary widely depending on the intensity of the field and duration of exposure. Some studies have shown that their use may increase biomass production, but their effects, which may be positive, null or even inhibitory, depend on the application conditions [12, 13]. Despite some advances, there are no studies of MF application to *N*. *oceanica* cultivation that consider both the growth and the induction of biomolecule synthesis.

Therefore, this study aims at investigating MF application to *N*. *oceanica* under different conditions and photoperiods to optimize production of biomass and compounds of biotechnological interest.

## Material and methods

### Microorganisms, cultivation media and conditions of the inoculum

Microalga *N*. *oceanica* **NANN OCEA-1** was provided by AlgaSul, a company in Rio Grande, RS, Brazil. The medium employed was f/2, as described by Andersen [14], containing 75 mg NaNO_3_, 5 mg Na_2_HPO_4_^.^H_2_O; 3.15 mg FeCl_3_^.^6H_2_O; 4.36 mg C_10_H_14_N_2_O_8_Na_2_^.^2H_2_O; 9.8 µg CuSO_4_^.^5H_2_O; 22 µg ZnSO_4_^.^7H_2_O; 10 µg CoCl_2_^.^6H_2_O; 180 µg MnCl_2_^.^4H_2_O; 6.3 µg Na_2_MoO_4_^.^2H_2_O; 100 µg C_12_H_17_ClN_4_OS^.^HCl; 0.5 µg C_63_H_88_CoN_14_O_14_P and 0.5 µg C_10_H_16_N_2_O_3_S per liter of saline water (28 g L^−1^). To prepare the inoculum, *N*. *oceanica* was kept in 250-mL Erlenmeyer flask with usable volume of 100 mL, at 25 °C, light incidence of 93 µmol m^−2^ s^−1^, photoperiod of 12L:12D, pH between 7–8 and salinity equal to 28 g L^−1^. No MF was applied on inoculum.

### Cultivation conditions

Cultivation was carried out in 1-L Erlenmeyer flasks with usable volume of 800 mL and kept at 25 ºC in an incubation chamber (ELETROlab EL202/3, Brazil) and agitation by aeration at 1.5 vvm, injecting sterile air through a sterilized glass wool filter. Two photoperiods were evaluated: 12L:12D for 15 d and 24L for 15 d with luminosity of 93 μmol photons m^−2^ s^−1^. Cultivation started at optical density (OD) of 0.1 measured at 750 nm, which corresponded to 1 × 10^6^ cells mL^−1^.

### MF application to *N*.* oceanica* cultivation

**Fig. 1:**
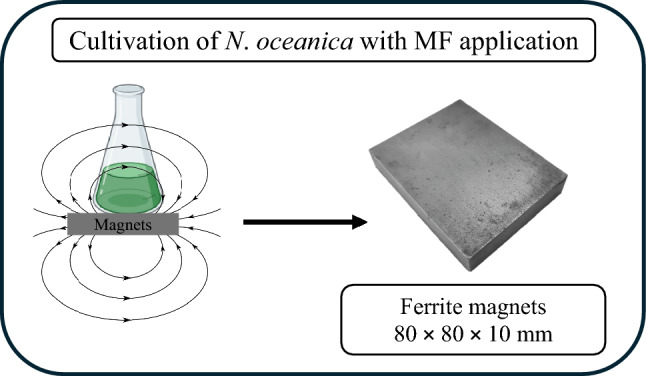
Schematic representation of magnetic field (MF) application during *N*. *oceanica* cultivation

Magnetic field (MF) were applied during the cultivation of *N*. *oceanica* by placing ferrite magnets (80 × 80 × 10 mm), with an average intensity of 30 mT, under the Erlenmeyer flasks. The average intensity of the MF inside the flasks was determined by taking at least 50 measurements with a Gaussmeter (model TLMP-HALL-05kT0, Brazil) to ensure greater spatial representativeness of the applied MF. Influence of length of MF application was evaluated throughout the whole cultivation period (24 h d^−1^) and for 1 h d^−1^. The control cultivation was also conducted in these conditions, but there was no MF application. A diagram of the experimental setup for the MF application can be seen in Fig. 1.

### Determination of pH and biomass concentration

Biomass concentration was quantified by a spectrophotometer (Visible spectrophotometer*,* V5000, China) at OD of 750 nm. It was calculated with the use of a standard curve (y (absorbance) = 2.5075 × (biomass concentration, g L^−1^) + 0.0595; R^2^ = 0.99), which related it to absorbance [21]. The pH determination was carried out in the cultivation supernatant by a digital pH meter (Quimis, Q400MT, Brazil).

For these analyses, 6 mL of samples were taken every day. This reduced the volume of the culture slightly throughout the experiment, but the variation remained within the acceptable margin of up to 20%. For collection, the aeration hoses were temporarily sealed to prevent contamination. Erlenmeyer flask was then taken close to the Bunsen burner to maintain a more sterile environment during the procedure, and there the 6 mL sample was taken using previously sterilized material. The entire process took a maximum of about 5 min. After that, the Erlenmeyer flasks returned to the aeration system inside the incubation chamber (ELETROlab EL202/3, Brazil), resuming normal experimental conditions.

### Growth parameters

The maximum specific growth rate (µmax, d^−1^) was calculated by linear regression in the logarithmic growth rate for each experiment, obtained from the plotted curve ln X_t_ versus t (d). The doubling/generation time (t_g_) was determined in the exponential growth cycle according to the equation (t_g_ = ln (2)/μ_max_ d^−1^).

### Recovery of biomass

At the end of cultivation (192 h), the entire volume of the culture medium containing the biomass was centrifuged (9690 × *g*; 20 °C; 20 min). The biomass was then washed in a 0.5 mol L^−1^ ammonium formate solution and centrifuged again. (9690 × *g*; 20 °C; 10 min) to enable salts to be removed. Afterwards, it was frozen at −80 °C for 48 h and lyophilized (−55 °C; 50 mmHg; 48 h).

### Biochemical composition of biomass

Lyophilized biomass was subject to determinations of moisture, proteins, carbohydrates, lipids and ashes. To determine carbohydrates and proteins, 10 mg biomass and 20 mL distilled water were sonicated (500 W; 20 kHz; 5 min) in an ice bath. Carbohydrates (%) were determined by the Dubois method [15]; absorbance was measured at 488 nm and a standard curve for glucose was performed. Proteins (%) were determined by the method proposed by Lowry et al. [16]; absorbance was measured at 750 nm and a standard curve for bovine serum albumin was performed.

Lipids (%) were extracted in agreement with Halim et al. [17] filtration (Whatman no. 1) and evaporation of the solvent. Moisture and ashes were determined at 105 °C and 550 °C for 3 h, respectively.

### Determination of pigment concentration

Pigment composition was determined by high performance liquid chromatography (HPLC), in agreement with Faé et al. [18]. The pigments were extracted from 50 mg freeze-dried biomass. Extraction was carried out with 3 mL buffered methanol 95% (2% ammonium acetate) containing 0.05 mg L^−1^ trans-β-Apo-8′-carotenal, followed by sonication in an ice bath for 5 min, incubation at −20 ºC for 1 h and centrifugation at 1.100 × g and 3 ºC for 5 min. The supernatant was filtered and mixed with 400 µL Milli-Q water before injection (1,000 µL) into the chromatographer (HPLC) with a monomeric C8 column and a pyridine mobile phase. Identification of pigments was based on absorption spectra and on retention times detected by a photodiode detector (Shimadzu SPDM20A, Japan).

### Determination of antimicrobial activity

Antimicrobial activity of extracts from *N*. *oceanica* biomass was evaluated against strains of *Acinetobacter baumannii* (ATCC 19606), *Pseudomonas aeruginosa* (ATCC 15442), *Vibrio coralliilyticus* (isolate) and *Staphylococcus aureus* (ATCC 12598), in agreement with protocol M07-A10 (CLSI, 2015). Biomass extracts at different concentrations (0.006–0.8 mg mL^−1^) were prepared in Mueller Hinton broth and inoculated with 5 × 10^5^ CFU mL^−1^. After incubation at 37 °C for 24 h, bacterial viability was determined by resazurin. Negative and positive controls were included while ceftazidime, ciprofloxacin and amikacin were the standards. All assays were carried out in triplicate.

### Determination of antioxidant activity

Determination of antioxidant activity was carried out in agreement with Lichtenthaler [19], with the use of methanolic extracts (25 mg lyophilized biomass in 10 mL methanol 99%, kept at −4 ºC for 24 h). Determination of scavenging of both radicals 2,2-diphenyl-1-picryl-hydrazyl (DPPH) and 2,2′-azino-bis(3-ethylbenzothiazoline-6-sulfonic) (ABTS) were conducted as proposed by Rufino et al. [20] In the DPPH assay, 0.1 mL extract (2.5 mg mL^−1^) was added to 3.9 mL DPPH (0.06 mmol L^−1^), kept in the dark for 60 min and read at 517 nm. Regarding ABTS, the radical cation was generated by the reaction between ABTS (7 mmol L^−1^) and potassium persulfate (140 mM) and diluted to absorbance of 0.70 ± 0.05 at 734 nm. Thirty μL extract was added to 3.0 mL solution and read after 6 min. Results were expressed as μmol Trolox g^−1^ of the sample.

### Statistical analysis

Responses of cultivation were evaluated by the analysis of variance (ANOVA, one-way), followed by the Tukey’s test and the Student’s t-test to determine antioxidant activity, at 95% confidence level (*p* < 0.05). All cultures and analytical measurements were performed in triplicate.

## Results and discussion

### Influence of MF on biomass growth and pH

**Fig. 2:**
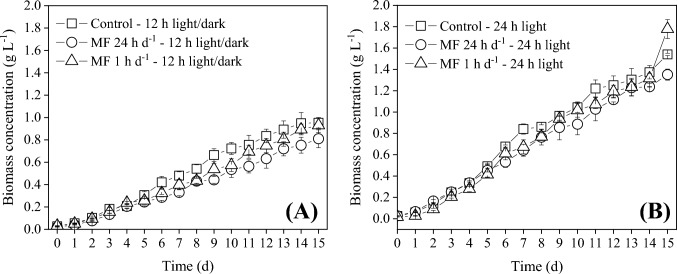
Biomass concentrations in* N*. *oceanica* cultures under 12L/12D/dark photoperiod (**A**) and 24L photoperiod (**B**) with MF application of 30 mT and control assays

*N. oceanica* exhibited growth in all conditions under investigation, as shown in Fig. 2. All cultures ended their exponential phase on the 15th day and reached maximum concentrations of biomass, like the ones described by Guerra et al. [21], who reported about 2.0 g L^−1^ after 15 days.

In the 12L/12D photoperiod (Fig. 2A), MF application of 30 mT, either for 1 h d^−1^ or 24 h d^−1^, did not result in significant statistical differences by comparison with the control. However, in continuous photoperiod (24L; Fig. 2B), there was high increase in biomass concentration, i. e., 62%, when application lasted 24 h on the field under 24L photoperiod, and 91%, when application lasted 1 h d^−1^ under 24L photoperiod. In all cases, the 15th day of cultivation was considered.

**Fig. 3:**
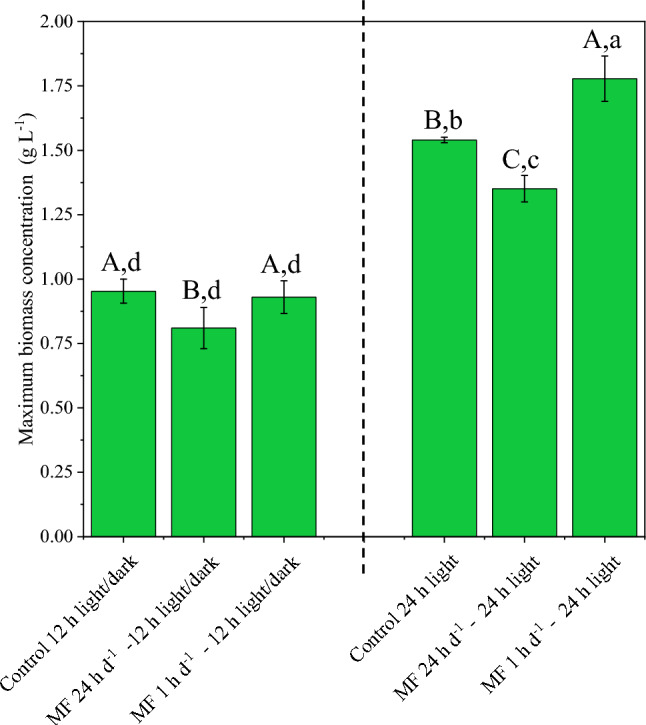
Maximum biomass concentrations of cultures (15 days) of *N*. *oceanica* under 12L/12D photoperiod and 24L with MF application of 30 mT. Different lowercase letters show significant difference (*p* < 0.05) among all assays while different uppercase letters show significant differences (*p* < 0.05) among equal photoperiod conditions

The highest biomass concentration was found when MF application lasted 1 h d^−1^ – 24L, followed by the control – 24-h light, and then MF application 24 h d^−1^ – 24L. Finally, in 12L/12D cultures (with and without any MF) (Fig. 3). This behavior is aligned with Menestrino et al. [22], who explained that MF application at the beginning of the light cycle coincides with the metabolic adjustment of cells, thus, favoring the synthesis of co-factors that are essential to photosynthesis, the activation of the transport system of electrons and the assimilation of metallic nutrients. Besides, effects of MF remain even after the exhibition, potentializing the use of continuous light and keeping the rate of cell growth high.

Growth kinetic parameters (Table 1) did not exhibit significant differences among treatments: µmax ranged between 0.085 ± 0.01 and 0.137 ± 0.05 d^−1^ while T_g_ ranged from 5.51 ± 1.86 to 8.24 ± 1.36 d. These values agree with the ones reported by Guerra et al*.* [21] (0.129 ± 0.020 d^−1^) but are lower than the ones reached by Khosravinia et al*.* [23] (0.25 ± 0.00 d^−1^). It is related to methodological differences, mainly regarding light intensity and length of cultivation.

**Table 1:** Growth parameters of *N*. *oceanica* under different photoperiods and MF application of 30 mT and control assays

Photoperiod	MF	µ_max_	T_g_
	(h d^−1^)	(d^−1^)	(d)
12L/12D	0	0.137 ± 0.05^Aa^	5.514 ± 1.86^Aa^
	1	0.130 ± 0.00^Aa^	5.324 ± 0.26^Aa^
	24	0.117 ± 0.00^Aa^	5.919 ± 0.45^Aa^
24L	0	0.085 ± 0.01^Aa^	8.249 ± 1.36^Aa^
	1	0.115 ± 0.01^Aa^	6.100 ± 0.84^Aa^
	24	0.119 ± 0.01^Aa^	5.896 ± 0.97^Aa^

^*^Generation time (T_g_), maximum rate of specific growth (µ_max_). Equal and lowercase letters mean that there is no significant difference among cultures while equal and uppercase letters on a certain column show that there is no significant difference in the same photoperiod, at 95% confidence level (*p* < 0.05)

**Fig. 4:**
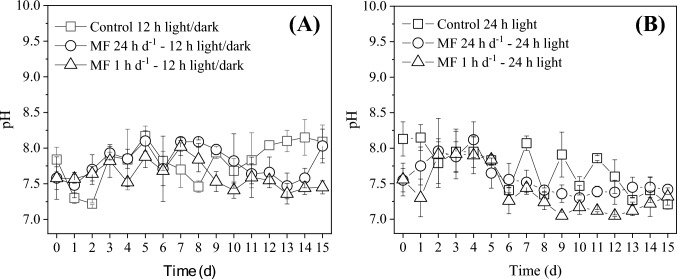
Behavior of pH in *N*. *oceanica* cultures under 24 h light (**A**) and 12 h light/dark photoperiod (**B**) with MF application of 30 mT and control assays

In cultures, pH (Fig. 4) ranged between 7.0 and 8.2 throughout the 15-day experiment. It is the ideal pH range for *N. oceanica* [24]. In the 12L/12D photoperiod (Fig. 4B), the initial pH (8.0) had a slight decrease up to 7.5 at the end of cultivation. In the 24L photoperiod (Fig. 4A), oscillations were from 7.5 to 8.0. The stability shows the balance between CO₂ assimilation and photosynthetic activity [25].

### Biomass characterization

Table 2 shows the proximate composition of biomass reached in different conditions. Carbohydrate content ranged between 23.70% and 36.73%. The highest values of carbohydrate content were achieved when MF were applied for 24 h d^−1^ – 12L/12D (36.73%) and the lowest ones when MF were applied for 1 h d^−1^ – 12L/12D (23.70%) and MF applied for 1 h d^−1^ – 24L (24.39%). These findings agree with the ones reported by Guerra et al. [21] and Senousy et al.[26], whose results ranged from 21 to 42% when *N*. *oceanica* grew in f/2 medium.

**Table 2:** Characterization of the biomass produced by *N*. *oceanica* under 12 h light/dark and 24 h light photoperiods with MF application of 30 mT (1 h d^−1^ or 24 h d^−1^) and control assays

Photoperiod	MF (h d^−1^)	Carbohydrates (%, w w^−1^)	Proteins (%, w w^−1^)	Lipids (%, w w^−1^)	Ashes (%, w w^−1^)
12L/12D	0	31.05 ± 1.93^ab^	31.58 ± 1.43^ab^	23.68 ± 0.94^c^	12.56 ± 2.11^b^
	1	23.70 ± 2.95^c^	32.23 ± 6.66^ab^	22.63 ± 1.14^c^	21.04 ± 2.99^a^
	24	36.73 ± 1.66^a^	14.80 ± 1.95^c^	31.53 ± 5.65^b^	15.26 ± 2.53^b^
24L	0	33.95 ± 1.45^ab^	36.43 ± 0.38^a^	23.90 ± 1.49^c^	4.52 ± 0.60^c^
	1	24.39 ± 3.07^c^	23.47 ± 4.65^ac^	46.45 ± 6.04ª	4.53 ± 0.47^c^
	24	30.47 ± 0.35^b^	29.48 ± 0.95^ab^	36.48 ± 1.91^b^	2.82 ± 0.66^c^

^*^Different letters on a column mean that the cultures are statistically equal; different letters on a column represent statistical difference at 95% confidence level

From a metabolic perspective, carbohydrates represent the primary photosynthetic products in microalgae and act as central carbon precursors for the synthesis of lipids and proteins [27, 28]. Through glycolysis and the tricarboxylic acid cycle, carbohydrates are oxidized and decreasing, generating acetyl-CoA and reducing equivalents that support fatty acid biosynthesis [29]. In parallel, carbon skeletons derived from carbohydrate metabolism are aminated and transaminated to form amino acids, which are subsequently polymerized into proteins [28]. Thus, lipid and protein accumulation are closely linked to carbohydrate turnover and respiratory activity [27, 30].

Protein contents ranged from 14.80% (MF 24 h – 12L/12D) to 36.43% (C – 24L). These values are in the range described by Ashour et al*.* [31] (14.46%) and Uthaiah et al. [32] (46.78%). The reduction in protein content under prolonged MF exposure (24L – 12L/12D) may indicate a shift in nitrogen assimilation or in the availability of carbon skeletons required for amino acid biosynthesis, possibly due to alterations in carbohydrate conversion dynamics.

Regarding lipids, the highest content was found in MF 1 h d-^1^ – 24L (46.45%), followed by MF 24 h d^−1^ – 24L (36.48%) and MF 24 h d – 12L/12D (31.53%). The increase under intermittent MF and continuous light suggests moderate stress induction which encourages lipid accumulation [33]. Considering that lipid biosynthesis depends on acetyl-CoA derived from carbohydrate catabolism, the elevated lipid content under intermittent MF may reflect a redistribution of carbon flux from carbohydrate storage toward fatty acid synthesis [29, 30]. Conversely, prolonged MF exposure associated with higher carbohydrate levels may indicate partial restriction of carbohydrate conversion, thereby limiting its channeling into lipid and protein biosynthesis [30].

Ash contents ranged between 2.82% (MF 24 h – 24L) and 21.04% (MF 1 h – 12L/12D). The high values reached in alternate photoperiods show that there was high mineral absorption, as observed by Souza et al. [34] and Kato et al. [35]. These results show that both the photoperiod and the regime of MF application influence carbon and energy allocation in cells, thus, guiding the metabolism to either lipid or carbohydrate accumulation, depending on the intensity and length of magnetic exposure.

Overall, the combined effects of photoperiod and MF regime appear to modulate carbon partitioning and metabolic flux distribution in the cells. The MF may interfere with enzymatic activity, electron transport processes, or redox balance [36, 37], thereby affecting carbohydrate utilization and its subsequent conversion into lipids and proteins [29]. This metabolic interplay helps explain the observed variations in macromolecular composition among treatments.

### Pigment content

The main pigments identified in *Nannochloropsis oceanica* at the end of 192 h of cultivation were chlorophyll-a, vaucheriaxanthin, violaxanthin, zeaxanthin, antheraxanthin, and β-carotene (Table 3).

**Table 3:** Pigment content of *N*. *oceanica* under different light conditions and MF application of 30 mT

Assays	Pigment content (mg g^−1^)^*^					
	Chlorophyll a	Vaucheriaxanthin	Violaxanthin	Zeaxanthin	Antheraxanthin	β-Carotene
Control—12L/12D	0.13 ± 0.04^a,b,c^	0.10 ± < 0.01^a,b^	0.06 ± 0.02^a^	0.01 ± < 0.01^a^	0.0040 ± 0.0010^a^	0.0004 ± 0.0002^b^
MF 24 h d^−1^—12L/12D	0.20 ± 0.03^a^	0.11 ± < 0.01^a^	0.07 ± 0.05^a^	0.01 ± < 0.01^a^	0.0036 ± 0.0003^a^	0.0017 ± 0.0005^a^
MF 1 h d^−1^—12L/12D	0.19 ± 0.09^a,b^	0.08 ± < 0.01^a,b^	0.04 ± 0.02^a^	0.01 ± < 0.01^a^	0.0034 ± 0.0021^a^	0.0004 ± 0.0001^b^
Control—24L	0.06 ± 0.03^b,c^	0.05 ± < 0.01^b^	0.03 ± 0.01^a^	0.01 ± < 0.01^a^	0.0029 ± 0.0026^a^	0.0001 ± 0.0002^b^
MF 24 h d^−1^—24L	0.05 ± 0.02^c^	0.06 ± < 0.01^a,b^	0.05 ± < 0.01^a^	0.01 ± < 0.01^a^	0.0022 ± 0.0004^a^	0.0003 ± 0.0002^b^
MF 1 h d^−1^—24L	0.06 ± 0.02^b,c^	0.06 ± < 0.01^a,b^	0.06 ± 0.02^a^	0.01 ± < 0.01^a^	0.0026 ± 0.0008^a^	0.0002 ± 0.0001^b^

^*^Different letters in the same column represent significant difference at a 95% confidence level (*p* < 0.05) between the assays

Chlorophyll-a contents ranged from 0.05 ± 0.02 mg g^−1^ (MF 24 h d^−1^ – 24L) to 0.20 ± 0.03 mg g^−1^ (MF 24 h d^−1^ – 12L/12D). The highest values were observed under the photoperiod condition (12L/12D) when MF were applied for 24 h d^−1^, suggesting that the combination of MF exposure and light/dark cycling may stimulate chlorophyll biosynthesis. Similarly, Bauer et al. (2017) obtained a 39.9% increase in chlorophyll-a production by the microalga *Chlorella kessleri* LEB 113 when they applied 60 mT for 1 h d^−1^ with a 12L/12D photoperiod. Deamici et al. (2018) demonstrated a 137.7% increase in chlorophyll-a content, of *Spirulina* sp. LEB 18, with the application of 25 mT 24 h d^−1^ and a 12L/12D photoperiod.

Violaxanthin contents ranged from 0.03 ± 0.01 mg g^−1^ (Control – 24L) to 0.07 ± 0.05 mg g^−1^ (MF 24 h d^−1^ – 12L/12D), with no significant differences among treatments (*p* > 0.05). Similarly, Zeaxanthin contents remained relatively constant across treatments, with values around 0.01 ±  < 0.01 mg g^−1^. Vaucheriaxanthin varied between 0.05 ±  < 0.01 mg g^−1^ (Control – 24L) and 0.11 ±  < 0.01 mg g^−1^ (MF 24 h d^−1^—12L/12D. Antheraxanthin concentrations ranged from 0.0022 ± 0.0004 mg g^−1^ (MF 24 h d^−1^ – 24L) to 0.0040 ± 0.0010 mg g^−1^ (Control – 12L/12D, with no significant differences observed among the assays (*p* > 0.05).

β-Carotene contents were the lowest among the pigments analyzed, ranging from 0.0001 ± 0.0002 mg g^−1^ (Control – 24L) to 0.0017 ± 0.0005 mg g^−1^ (MF 24 h d^−1^ – 12L/12D). Although most treatments did not differ significantly, the MF 24 h d^−1^ – 12L/12D condition resulted in the highest β-carotene accumulation.

Overall, pigment accumulation tended to be higher under the 12L/12D photoperiod, particularly when MF were applied continuously (24 h d^−1^), indicating a possible interaction between MF exposure and photoperiod in regulating carotenoid and chlorophyll biosynthesis in *N*. *oceanica*.

### Antimicrobial activity

Evaluation of antimicrobial activity in *Nannochloropsis oceanica* is relevant due to the increasing interest in microalgae as sources of bioactive compounds with potential applications in animal production [38] and aquaculture industries [39]. This species is known to produce bioactive metabolites, particularly phenolic compounds, fatty acids and other lipophilic compounds, which have been associated with antimicrobial activity in microalgae [40]. Therefore, assessing the antimicrobial potential of its biomass contributes to exploring its biotechnological value and possible future applications.

Biomass produced by *N. oceanica* did not exhibit significant antimicrobial activity against *Acinetobacter baumannii*, *Pseudomonas aeruginosa*, *Vibrio coralliilyticus* and *Staphylococcus aureus*. The absence of effect is attributed to the high dilution of the sample and to the use of biomass, instead of the supernatant, where bioactive extracellular extracts usually accumulate [41]. However, previous studies reported that methanolic extracts of this species exhibit moderate antimicrobial activity against *S*. *aureus* and *E*. *coli* [42].

### Antioxidant activity

Assays ABTS⁺ and DPPH (Table 4) indicated moderate antioxidant activity. Inhibition was 20.2 ± 3.14% and 22.3 ± 0.49% (MF 1 h d^−1^ – 12L/12D) and 16.0 ± 6.86% and 23.8 ± 0.73% (MF 1 h d^*−*1^ – 24L), respectively. There was no significant difference among photoperiods (*p* < 0.05). These results agree with the ones reported by Conde et al. [43], who observed about 20% inhibition of radicals and confirmed the presence of antioxidant compounds in *N. oceanica*.

**Table 4:** ABTS and DPPH free radical scavenging activity of *N*. *oceanica* cultivated under a 30 mT (1 h d^−1^) under two photoperiod regimes: 24L and 12L/12D

Assays	DPPH (%)	ABTS^+^ (%)
MF 1 h d^−1^ – 12L/12D	20.2 ± 3.14^a^	22.3 ± 0.49^a^
MF 1 h d^−1^ – 24L	16.0 ± 6.86^a^	23.8 ± 0.73^a^

^*^Equal letters on a certain column show that there was no statistical difference by the t-test at 95% confidence level (*p* < 0.05)

## Conclusion

Application of 30 mT (MF) modulated the biochemical composition of *Nannochloropsis oceanica* without significantly affecting growth rate. However, the condition MF 1 h d^−1^ under continuous light (24L) resulted in the highest biomass concentration and lipid accumulation (46.45%), indicating impro**v**ed carbon allocation toward lipid biosynthesis. Pigment accumulation, particularly chlorophyll *a*, violaxanthin, and vaucheriaxanthin, was also supported under this condition. Overall, short-term MF exposure combined with continuous illumination appears to be the most favorable strategy to enhance biomass productivity and the production of lipids and high-value pigments in *N*. *oceanica*.

## Data Availability

All data generated and analyzed by this study are included in this manuscript (and in its supplementary material). The data can be made available.
